# The CRB1 Complex: Following the Trail of Crumbs to a Feasible Gene Therapy Strategy

**DOI:** 10.3389/fnins.2017.00175

**Published:** 2017-04-05

**Authors:** Peter M. Quinn, Lucie P. Pellissier, Jan Wijnholds

**Affiliations:** ^1^Department of Ophthalmology, Leiden University Medical CenterLeiden, Netherlands; ^2^Unité Physiologie de la Reproduction et des Comportements, INRA UMR85, Centre National de la Recherche Scientifique UMR-7247, Institut Français du Cheval et de l'Équitation, Université François RabelaisNouzilly, France; ^3^Netherlands Institute for Neuroscience, Royal Netherlands Academy of Arts and SciencesAmsterdam, Netherlands

**Keywords:** crumbs complex, retinopathies, gene therapy, CRISPR, human iPSC, retinal organoids

## Abstract

Once considered science fiction, gene therapy is rapidly becoming scientific reality, targeting a growing number of the approximately 250 genes linked to hereditary retinal disorders such as retinitis pigmentosa and Leber's congenital amaurosis. Powerful new technologies have emerged, leading to the development of humanized models for testing and screening these therapies, bringing us closer to the goal of personalized medicine. These tools include the ability to differentiate human induced pluripotent stem cells (iPSCs) to create a “retina-in-a-dish” model and the self-formed ectodermal autonomous multi-zone, which can mimic whole eye development. In addition, highly specific gene-editing tools are now available, including the CRISPR/Cas9 system and the recently developed homology-independent targeted integration approach, which allows gene editing in non-dividing cells. Variants in the *CRB1* gene have long been associated with retinopathies, and more recently the *CRB2* gene has also been shown to have possible clinical relevance with respect to retinopathies. In this review, we discuss the role of the CRB protein complex in patients with retinopathy. In addition, we discuss new opportunities provided by stem cells and gene-editing tools, and we provide insight into how the retinal therapeutic pipeline can be improved. Finally, we discuss the current state of adeno-associated virus-mediated gene therapy and how it can be applied to treat retinopathies associated with mutations in *CRB1*.

## *CRB1*-related retinopathies: no clear phenotype-to-genotype correlation

*CRB1*-linked retinal dystrophies represent a diverse spectrum and present with a wide complexity of clinical features (Table 1). In children, mutations in the *CRB1* gene have been identified as a causal factor underlying Leber's congenital amaurosis (LCA) and early-onset retinitis pigmentosa (RP) (den Hollander et al., 1999; Richard et al., 2006). The *CRB1* gene has been linked to 7–17% of autosomal recessive LCA cases and 3–9% of autosomal recessive RP cases (Vallespin et al., 2007; Bujakowska et al., 2012; Corton et al., 2013). In patients, *CRB1*-linked LCA is associated with atypical thickening of the retina and disorganized retinal layering (Jacobson et al., 2003; Aleman et al., 2011). Both of these features are also present in double-knockout mice lacking both *Crb1* and *Crb2* in their retinal progenitor cells. During development, these *Crb1Crb2* double-knockout mice also have dysregulated apical-basal polarity in the retina, altered retinal progenitor cell proliferation, and reduced downstream CRB signaling, including dysregulation of YAP (Yes-associated protein). These findings highlight the essential role that the CRB (Crumbs) complex plays in normal retinal development (Pellissier et al., 2013).

**Table 1:** **Summary of patient phenotypes associated with mutations in the ***CRB1*** gene**.

**Phenotype**	**Inheritance**	**References**
Leber congenital amaurosis 8 (LCA8)	AR	Jacobson et al., 2003; Cordovez et al., 2015; Talib et al., in press
Early-onset retinitis pigmentosa (RP)	AR	den Hollander et al., 1999; Lotery et al., 2001
RP with preserved para-arteriolar retinal pigment epithelium	AR	Heckenlively, 1982
RP with intraretinal cystoid spaces	AR	Cordovez et al., 2015
RP with Coats-like exudative vasculopathy	AR	den Hollander et al., 2001
Peripheral nummular pigmentation	AR	Bujakowska et al., 2012
Pigmented paravenous chorioretinal atrophy	AD	McKay et al., 2005
Cystoid macular edema	AR	Morarji et al., 2016; Tsang et al., 2014
Macular atrophy	AR	Bujakowska et al., 2012
Familial foveal retinoschisis	AR	Vincent et al., 2016

*AD, autosomal dominant; AR, autosomal recessive*.

More than 230 pathogenic variants have been identified in the *CRB1* gene (see http://exac.broadinstitute.org/transcript/ENST00000367400 and http://databases.lovd.nl/shared/variants/CRB1). It is not currently clear why a given variant can lead to either early-onset LCA or RP within the disease spectrum. A possible modifier of this effect in the human retina is CRB2, as shown in the mouse retina (Pellissier et al., 2014b). Early studies suggest that variants in the *CRB2* gene are not a frequent cause of either autosomal recessive LCA or RP (van den Hurk et al., 2005). However, missense mutations in the human *CRB2* gene were recently associated with minor retinal symptoms, including mild optic atrophy, reduced visual acuity, and irregular retinal pigmentation, in a subset of patients (Lamont et al., 2016). Interestingly, the *CRB2* gene is also expressed in vital organs such as the brain, testis, and kidney, and genetic variants lead to a clinically extensive syndromic phenotype causing multiple abnormalities and lethality (Lamont et al., 2016). Homozygous and/or heterozygous variants are reported to cause brain conditions (e.g., ventriculomegaly and hydrocephalus), kidney conditions (e.g., congenital nephrosis, steroid-resistant nephrotic syndrome, and ureteropelvic renal anomalies), and other conditions such as lung hypoplasia and cardiac malformation (Ebarasi et al., 2015; Slavotinek et al., 2015; Jaron et al., 2016; Lamont et al., 2016).

*Crb2* knockout mice are embryonic lethal due to a defect in epithelial-to-mesenchymal transition during the gastrulation stage (Xiao et al., 2011; Ramkumar et al., 2016). In addition, proteins that modify the extracellular domain of Crb2 (for example, O-glucosyltransferase-1) can alter the receptor's function (Ramkumar et al., 2015). The offspring of conditional *Crb2* knockout mice crossed with Crx*Cre* mice mimic the human *CRB1*-linked RP phenotype and develop hydrocephalus (Alves et al., 2014a). Consistent with this report, conditionally knocking out YAP—a Hippo pathway effector and an interactor with CRB complex members—was recently reported to cause hydrocephalus in a mouse model due to a disruption in the CRB complex and adherens junctions (Varelas et al., 2010; Bui et al., 2016; Park et al., 2016). Finally, although *CRB3* mRNA has been found in the macula and peripheral retina, the *CRB3* gene has yet to be linked to retinal disease (Pellissier et al., 2014b).

## CRB expression and localization

**Figure 1:**
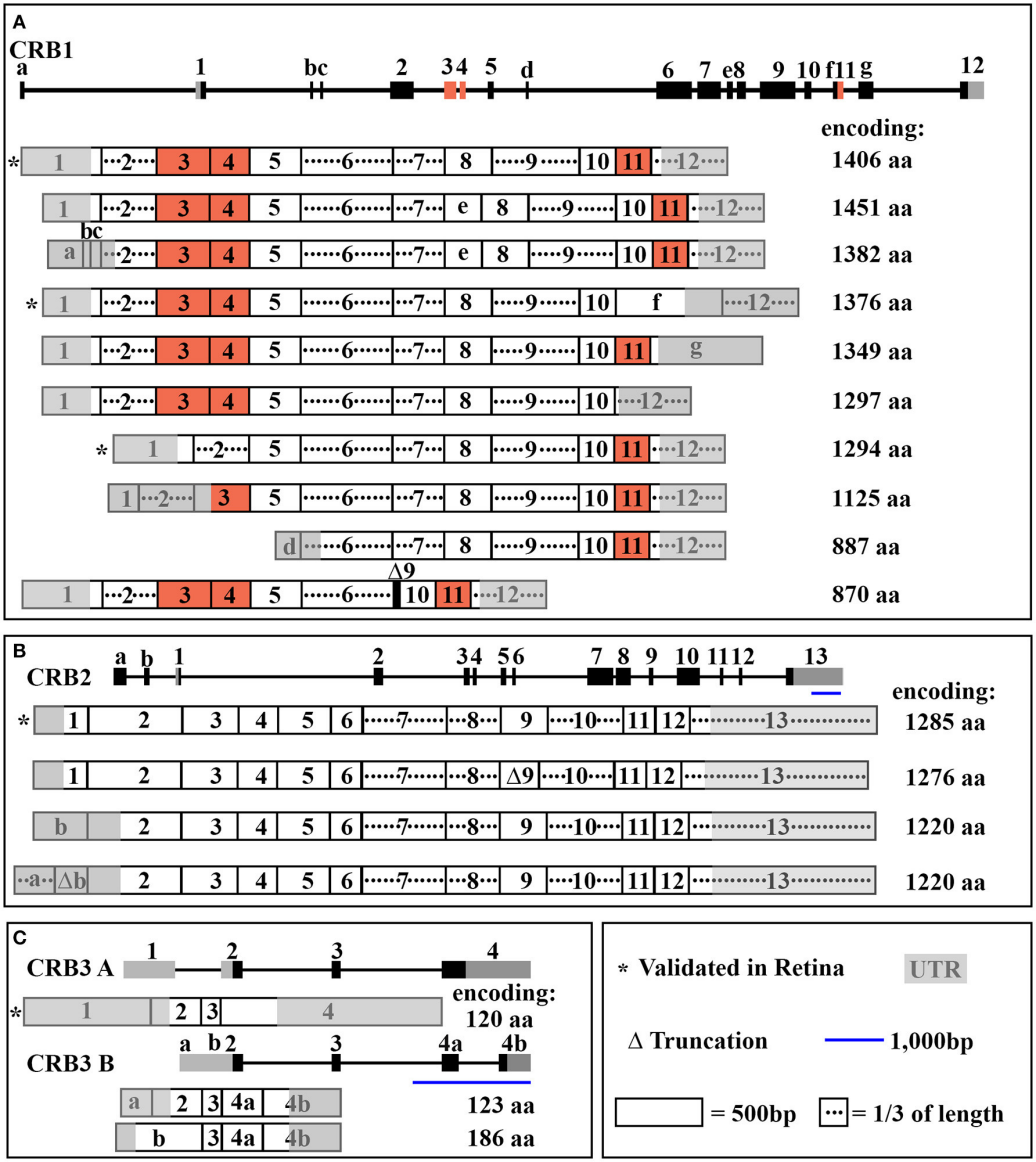
**Schematic overview of ***CRB*** transcripts. In each panel, the gene structure is shown at the top, with the exons indicated. (A)** The entire *CRB1* gene with exons 1–12, alternative exons a through g, and the 10 predicted mRNA transcript variants that encode their respective protein isoforms. **(B)** The entire *CRB2* gene with exons 1-13, alternative exons a and b, and the four predicted mRNA transcript variants that encode their respective protein isoforms. **(C)** The entire *CRB3* gene with exons 1-4, alternative exon a, b, 4a, and 4b, and the two mRNA transcript variants (*CRB3A* and *CRB3B*) that encode their respective protein isoforms. See Table 2 for further details.

**Figure 2:**
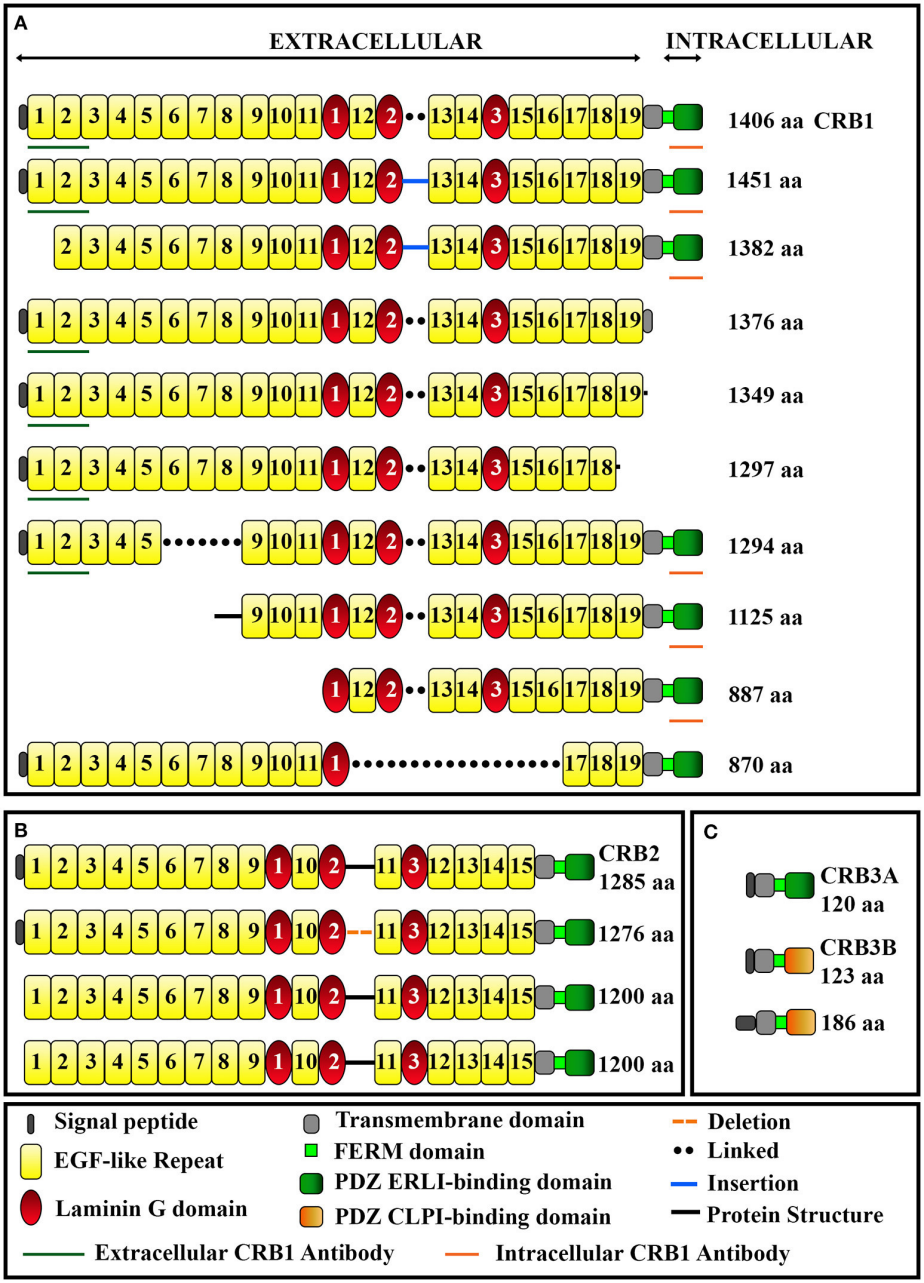
**Schematic overview of the domains present in CRB1 (A)**, CRB2 **(B)**, and CRB3 **(C)** protein isoforms. The epitopes for the extracellular and intracellular anti-CRB1 antibodies are also indicated. See Table 2 for further details.

The human *CRB1* gene is a complex, large gene mapped to chromosome 1q31.3. The gene contains 12 exons spanning 210 kb of genomic DNA (Figure 1A and Table 2) (den Hollander et al., 1999, 2004). The gene has 10 predicted transcript variants, 95 orthologs, and 10 paralogs (interestingly, these are involved primarily in Notch signaling) (http://www.ensembl.org/Homo_sapiens/Gene/Summary?db=core;g=ENSG00000134376;r=1:197268204-197478455). To date, mRNA corresponding to three *CRB1* transcript variants has been identified in the retina (Figure 1A, Table 2); these variants are expressed at similar levels in the macula and periphery of the retina, but are below detectable levels in adult retinal pigment epithelium and choroid tissues (Pellissier et al., 2014b). The first validated transcript variant contains 12 exons and encodes the prototypic canonical CRB1 isoform. This 1406-aa protein contains a signal peptide, 19 epidermal growth factor-like domains, 3 laminin-A globular domains, a single C-type lectin domain, a single transmembrane domain, and a short (37-aa) intracellular domain (Figure 2A; den Hollander et al., 2004). In contrast, the second validated transcript, which encodes a 1376-aa isoform of CRB1, contains an alternative exon 11 (exon f; see Figure 1A). This isoform lacks the transmembrane and intracellular domains, possibly serving as a putative secreted protein (Figure 2A; den Hollander et al., 1999). The third validated transcript encodes a 1294-aa isoform of CRB1; this transcript lacks exons 3 and 4, causing the in-frame deletion of epidermal growth factor-like domains 6 through 8 while retaining both the N- and C-termini present in the prototypic CRB1 isoform (Figures 1A, 2A). Another alternatively spliced transcript encodes a 1382-aa isoform of CRB1. This transcript contains 15 exons: an additional exon (exon e) lies between exons 7 and 8, and the prototypic first exon is replaced by three noncoding exons (exons a, b, and c) in the 5′ UTR, resulting in a protein with a shorter N-terminus (Figures 1A, 2A).

**Table 2:** **Overview of CRB1, CRB2, and CRB3 isoforms and transcript variants**.

**Gene**	**Protein (with GenBank accession number and primary structure relative to the respective prototypic isoform)**		**Transcript Variant (with GenBank accession number and primary structure relative to the respective prototypic transcript)**		**Transcript Validated in the Retina**
*CRB1* chromosome 1 276,993 bp 12 exons	1,451 aa XP_016856341.1	45-aa insertion between laminin G-like 2 and EGF-like 13 domains.	XM_017000852.1	Contains an alternative in-frame exon (exon e).	unknown
	1,406 aa (CRB1) NP_957705.1	Prototypic isoform.	NM_201253.2	Prototypic transcript.	Validated (den Hollander et al., 2001; Pellissier et al., 2014b)
	1,382 aa NP_001244894.1	69-aa deletion resulting in loss of EGF-like 1 and a 45-aa insertion between laminin G-like 2 and EGF-like 13 domains compared to the prototypic isoform. Loss of signal peptide.	NM_001257965.1	Alternative transcription start site. Three noncoding exons (exons a, b, and c) in place of the first exon. Contains an alternative in-frame exon (exon e).	unknown
	1,376 aa AAL10681.1	First 1335 aa match the prototypic isoform, with an additional 41 aa at the C-terminus. Truncation of intracellular domain.	AY043324.1	Uses an alternative splice junction at the 3′ end in the coding exon (exon f) containing a stop codon.	Validated (den Hollander et al., 2001; Pellissier et al., 2014b)
	1,349 aa XP_011507667.1	First 1335 aa match the prototypic isoform, with an additional 14 aa. Truncation of intracellular domain.	XM_011509365.2	Alternative coding exon (exon g) containing a stop codon.	unknown
	1,297 aa XP_011507669.1	First 1292 aa match the prototypic isoform. Loss of the intracellular domain and EGF-like 19.	XM_011509367.1	Exon 11 deleted. Alternative stop codon in exon 12.	unknown
	1,294 aa NP_001180569.1	First 217 aa and last 1077 aa match the prototypic isoform. In-frame deletion of 112-aa (EGF-like domains 6-8).	NM_001193640.1	Exons 3 and 4 deleted.	Validated (Pellissier et al., 2014b)
	1,125 aa XP_016856340.1	The last 1076 aa match the prototypic isoform. Loss of EGF-like domains 1-8. Alternative translation start.	XM_017000851.1	Alternative transcription start site in exon 3. Loss of coding exon 4.	unknown
	887 aa XP_011507671.1	The last 887 aa math the prototypic isoform. Loss of EGF-like domains 1-11 and the signal peptide.	XM_011509369.2	Loss of coding exons 1-5. Contains additional noncoding exon (exon d). Alternative transcription start site in exon 6.	unknown
	870 aa NP_001244895.1	The first 709 aa and the last 161 aa match the prototypic isoform. Loss of laminin G-like 2 and 3 and EGF-like domains 12-16.	NM_001257966.1	Loss of two coding exons (exons 7 and 8) and most of exon 9.	unknown
CRB2 chromosome 9 25,876 bp 13 exons	1,285 aa (CRB2) NP_775960.4	Prototypic and longest isoform.	NM_173689.6	Prototypic transcript.	Validated (Pellissier et al., 2014b)
	1,276 aa XP_011516858.1	9-aa deletion between EGF-like 11 and laminin G-like 2.	XM_011518556.2	Truncation of exon 9.	unknown
	1,220 aa XP_011516859.1	Deletion of the first 65 aa. Loss of signal peptide.	XM_011518557.2	Loss of exon 1. Alternative noncoding exon (exon b). Alternative translation start site in exon 2.	unknown
	1,220 aa XP_011516860.1	Deletion of the first 65 aa. Loss of signal peptide.	XM_011518558.2	Loss of exon 1. Alternative noncoding exons (exons a and b). Alternative translation start site in exon 2.	unknown
CRB3 Chromosome 19 3,444 bp 4/5 exons	120 aa (CRB3A) NP_777378.1	Prototypic isoform with ERLI motif in the PDZ-binding domain.	NM_174882.2	Prototypic transcript.	Validated (Pellissier et al., 2014b)
	123 aa (CRB3B) NP_777377.1	Alternative PDZ-binding domain (CLPI).	NM_174881.3	Exon 1 replaced with alternative exon a. Uses alternative splice acceptors and splice donors in exon 4 to generate exons 4a and 4b.	Not detected in adult human retina and RPE (Pellissier et al., 2014b)
	186 aa XP_016882960.1	Alternative PDZ-binding domain (CLPI).	XM_017027471.1	Exons 1 and 2 replaced with alternative exon b. Translation start site in exon b. Uses alternative splice acceptors and splice donors in exon 4 to generate exons 4a and 4b.	unknown

**Figure 3:**
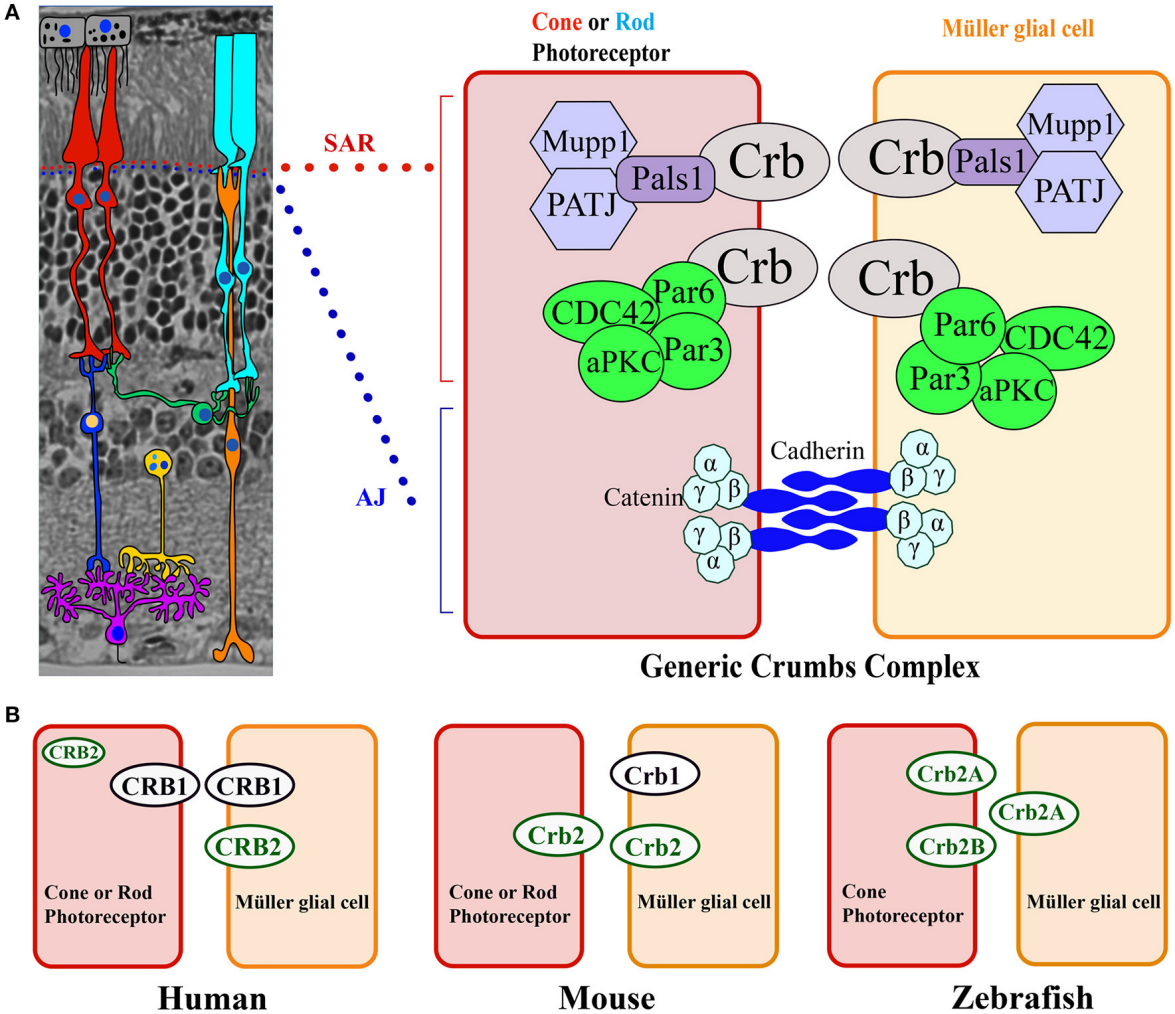
**Model of the CRB complex in the retina in general, as well as in the human, mouse, and zebrafish retina. (A)** General structure of the retina, which is composed of seven cell types: Müller glial cells (orange), bipolar cells (dark blue), horizontal cells (green), amacrine cells (yellow), retinal ganglion cells (purple), rods (light blue), and cones (red). The cell types are depicted over an image of a mouse section embedded in Technovit resin. The Crumbs complex is localized at the subapical region (SAR) above the adherens junction (AJ) between photoreceptors, between Müller glial cells, and between photoreceptor and Müller glial cells. At the right, the proteins that comprise the Crumbs complex and adherens junctions are shown schematically. **(B)** Model depicting CRB protein localization in photoreceptors and Müller glial cells in the human, mouse, and zebrafish retina.

In mammals, CRB1 is one of a three-member family of CRB proteins, together with CRB2 and CRB3. In humans, both CRB2 and CRB3 have additional predicted transcript variants that encode various protein isoforms in humans (Figures 1B–C, 2B–C, and Table 2). Both CRB1 and CRB2 contain a large extracellular domain with epidermal growth factor-like domains and laminin-A globular domains. The *CRB3* gene encodes two isoforms (CRB3A and CRB3B), both of which lack an extracellular domain (Bulgakova and Knust, 2009). In addition, the prototypic CRB1, CRB2, and CRB3A proteins contain a single transmembrane domain and a short, highly conserved 37-aa intracellular domain, a FERM (4.1, ezrin, radixin, moesin) domain juxtaposed with the transmembrane domain, and a C-terminal PDZ-binding motif. The 4-aa ERLI (Glu-Arg-Leu-Ile) sequence in the C-terminal PDZ domain is important for the protein's interaction with key adaptor proteins, including PALS1 and PAR6 (Klebes and Knust, 2000; Bachmann et al., 2001; Lemmers et al., 2004). Binding of PALS1 to the C-terminal PDZ domain leads to the recruitment of PATJ and MUPP1 and the assembly of the core CRB complex. Binding of PAR6 to the C-terminal PDZ domain leads to the recruitment of PAR3, aPKC (atypical protein kinase C), and CDC42, known as the PAR complex (Figure 3A; Hurd et al., 2003; Bulgakova and Knust, 2009). Via these proteins, the CRB complex regulates apical-basal polarity, modulates apical membrane size, and maintains cell adhesion through the cadherin-catenin complex at adherens junctions (Hsu et al., 2006; Laprise et al., 2006; Gosens et al., 2007; Gamblin et al., 2014). The FERM-binding domain—which sits adjacent to the PDZ domain—binds other proteins such as EPB4.1L5, which plays a role in the epithelial-to-mesenchymal transition in the gastrulation stage of development (Lee et al., 2007; Hirano et al., 2008). Although the function of EPB4.1L5 in the mammalian retina is not currently known, in zebrafish this protein plays a role in retinal development and is a putative negative regulator of outer segment size in rod photoreceptors (Christensen and Jensen, 2008). Binding of PDZ and FERM proteins to their respective binding motifs in CRB is mutually exclusive (Li et al., 2014; Wei et al., 2015), suggesting that different CRB complexes may exist, each with a specific function. Consistent with this hypothesis, the PDZ domain in the non-prototypic CRB3B isoform contains a C-terminal CLPI (Cys-Leu-Pro-Ile) motif instead of an ERLI motif (Figure 2C), and CRB3B plays a role in ciliogenesis and cell division (Fan et al., 2007).

**Figure 4:**
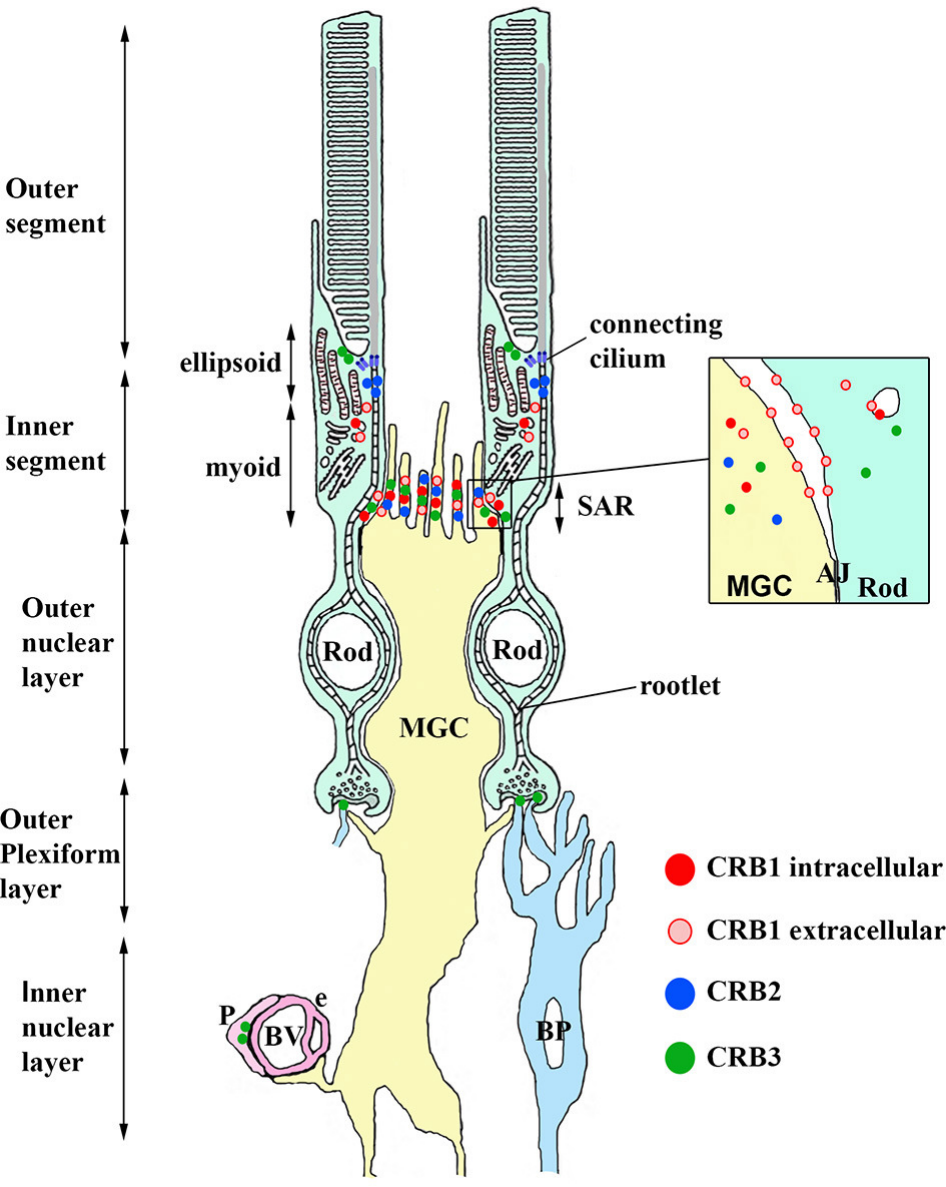
**Model depicting the localization of CRB1, CRB2, and CRB3 proteins in retinal cells and structures**. CRB1, detected using the intracellular CRB1 antibody (dark red) and extracellular CRB1 antibody (light red), is present in both Müller glia cells (MGC) and photoreceptor cells at the subapical region (SAR) above the adherens junctions (AJ, shown in the inset). CRB2 (blue) is present in MGCs at the SAR above the AJ. CRB3 (green) is present at the SAR in MGCs and photoreceptors. CRB3 is also present in the ellipsoid region of the inner segment, in the dendrites of rod bipolar (BP) cells, and in pericytes (P) in the blood vessels (BV). See the text for further details.

CRB proteins are localized primarily at the subapical region above the adherens junctions between two or more photoreceptors, between two or more Müller glial cells, and between photoreceptors and Müller glial cells (Figures 3A, 4; Pellikka et al., 2002; van de Pavert et al., 2004; Kantardzhieva et al., 2005). In the subapical region, human CRB1 is present in the microvilli of Müller glial cells and in the inner segments of photoreceptor cells (Figure 4). Interestingly, an antibody that recognizes all isoforms of CRB1 containing the prototypic N-terminus (the “extracellular CRB1” antibody; see Figures 2A, 4) reveals the presence of CRB1 proteins along the membranes of photoreceptors and Müller glial cells; in contrast, an antibody against the intracellular domain of CRB1 (the “intracellular CRB1” antibody) shows only patchy or vesicular staining (Pellissier et al., 2015). This difference in localization patterns may be due to the presence of the secreted 1376-aa form of CRB1 (Figures 2A, 4). In addition to its localization at the subapical region, CRB1 is also localized at vesicles in the vicinity of mitochondria throughout the myoid region of the inner segments of both rods and cones. Finally, CRB1 is also present in the outer plexiform layer of Müller glial cells, surrounding photoreceptor axons in Henle's fiber structure at the fovea (Figure 4).

In the human retina, CRB2 is localized in Müller glial cells (specifically, at the subapical region) and photoreceptor inner segments (in vesicles, presumably in the striated ciliary rootlets at the apical tips known as the ellipsoid region) (Figure 4; Pellissier et al., 2015). CRB3 is present at the subapical region in the microvilli of Müller glial cells and in the inner segments of photoreceptor cells. In addition, CRB3 is localized in the ellipsoid region at the interface between inner and outer segments. In the outer plexiform layer, CRB3 is localized to the dendrites of rod bipolar cells and in vascular pericytes (Figure 4; Pellissier et al., 2014b, 2015).

CRB proteins are conserved among species and have both overlapping and compensatory roles and functions (Pellissier et al., 2015). In the human retina, CRB1 is located at the subapical region in both Müller glial and photoreceptor cells, whereas CRB2 is located exclusively at the subapical region in Müller glial cells. CRB1, CRB2, and CRB3A are all present in the inner segments of photoreceptors in specific, delimited patterns. Surprisingly, the mouse retina has the opposite localization pattern at the subapical region (Figure 3B; van de Pavert et al., 2004; van Rossum et al., 2006). In zebrafish, Crb1 is not present at the subapical regions of photoreceptors and Müller glial cells; instead, two isoforms of Crb2—Crb2A and Crb2B—are present (Figure 3B; Zou et al., 2012). Interestingly, when human CRB2 is expressed selectively in mouse photoreceptors that lack endogenous Crb2, it also localizes to the tip of inner segments, presumably at striated ciliary rootlets. In contrast, when expressed in mouse photoreceptors and Müller glial cells, human CRB2 localizes to the subapical region (Pellissier et al., 2015). Previous studies showed that in both zebrafish and mice, Crb2 plays a role in determining the segment length of photoreceptors (Hsu and Jensen, 2010; Alves et al., 2013b). Moreover, CRB proteins may play complementary roles in photoreceptor inner segments. For example, in *Drosophila* myosin V is essential for transporting rhodopsin, and CRB stabilizes myosin V in order to mediate this transport (Pocha et al., 2011).

## Moving from animal models to the laboratory dish

**Figure 5:**
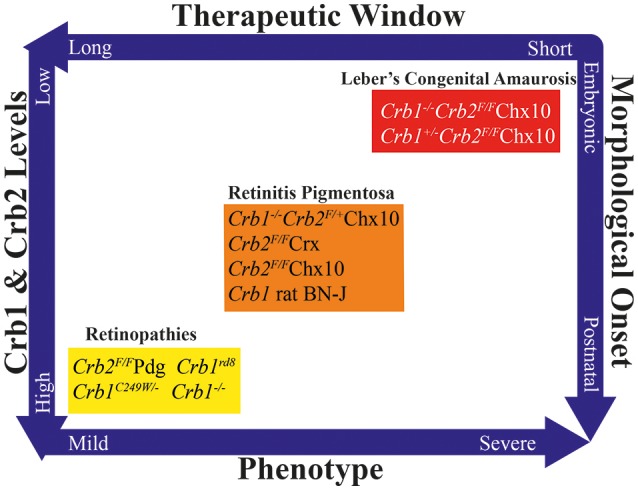
**Overview of CRB animal models, showing the duration of the putative therapeutic window, total Crb1 and Crb2 levels, phenotype severity, and timing of morphological onset**. Based on this multidimensional pattern, the various models can be grouped into models that develop mild retinopathies, early-onset retinitis pigmentosa, or Leber's congenital amaurosis.

Recent analyses of mammalian models of *CRB1*-linked retinal diseases provided key insight into the role of CRB proteins in the retina. A variety of models are now available for studying the function of both mutant *Crb1* and mutant *Crb2* (Figure 5). These models mimic the diverse phenotypes and severities observed in patients with *CRB1*-linked retinal dystrophies, including LCA, early-onset RP, telangiectasia, and mild retinopathies (van de Pavert et al., 2004; Alves et al., 2013b; Pellissier et al., 2013, 2014b; Zhao et al., 2015). These models have also provided clues to the cellular and molecular mechanisms that underlie the downstream actions of CRB1 and CRB2 (van de Pavert et al., 2007a; Pellissier et al., 2013; Alves et al., 2013a). Models that mimic mild retinopathies include the Crb1-knockout (*Crb1*^−/−^) mouse, the *Crb1*^*C*249*W*/−^ knock-in mouse, the naturally occurring *Crb1*^*rd*8^ mouse, and the Müller glial cell-specific *Crb2*Pdgfrα*Cre* knockout mouse (Mehalow et al., 2003; van de Pavert et al., 2004, 2007b; Alves et al., 2014a). All these models have several features in common, including loss of integrity at the subapical region-adherens junctions at the outer limiting membrane, displaced photoreceptors in the subretinal space, and focal upregulation of glial fibrillary acidic protein (GFAP). *Crb2*Chx10*Cre* and *Crb2*Crx*Cre* cell-specific knockout mice (which lack Crb2 in retinal progenitor and photoreceptor cells, respectively) and *Crb1Crb2*^*F*/+^Chx10*Cre* double-knockout mice (which lack Crb1 and have a 50% reduction in Crb2 levels) develop an early-onset RP phenotype (Alves et al., 2013a,b, 2014a; Pellissier et al., 2013). The BN-J rat (a mutant line of Brown Norway rat with a mutation in *Crb1*) develops an early-onset RP phenotype and telangiectasia (Zhao et al., 2015). These more severe rodent models develop photoreceptor half-rosettes in the outer nuclear layer and relatively early-onset photoreceptor degeneration. The double-knockout *Crb1Crb2*Chx10*Cre* mouse (which lacks both *Crb1* and *Crb2* in retinal progenitor cells) develops LCA; the double-knockout *Crb1*^+/−^*Crb2*Chx10*Cre* mouse (which lacks one allele of *Crb1* and both alleles of *Crb2* in retinal progenitor cells) also develops LCA (Pellissier et al., 2013). These models are characterized by an early-onset severe reduction in retinal activity (measured using electroretinography), a loss of photoreceptor inner and outer segment layers, a loss of the outer plexiform layer, fusion between the outer and inner nuclear layers, and ectopic retinal cells in all nuclear layers.

These three phenotypically distinct sets of mutant *CRB* models highlight the important role that CRB proteins play in various cell types throughout life (Figure 5). These models also illustrate that the total amounts of CRB proteins expressed in various cell types can strongly influence the severity of the phenotype (Alves et al., 2014b; Pellissier et al., 2014b). For example, a mild decrease in CRB levels leads to a relatively milder form of retinopathy, whereas greater reductions in CRB1 and CRB2 lead to early-onset RP; finally, a complete lack of CRB1 and CRB2 leads to LCA. These reductions in CRB levels also lead to variations in morphological onset: postnatally, late or early embryonically respectively. In turn, this correlates to the duration of the therapeutic window. Currently, the most suitable models for use in preclinical studies are the mouse models that develop early-onset RP, as these models most closely mimic human retinopathies with early-onset retinal degeneration. Whether the neurodevelopmental retinal disorganization present in LCA can be improved using gene therapy—and whether retinal organization can be restored by restoring CRB levels—is currently unknown. The therapeutic window for preventing the phenotype in mouse models of CRB1-associated LCA suggests that *in utero* application is needed for introducing gene therapy vectors. In order to demonstrate proof-of-concept with respect to this neurodevelopment-based phenotype, viral vectors will require further development, for example using specific promoters and/or AAV serotypes. This approach would facilitate the targeting and expression of CRB proteins during retinal development and maturation.

The animal models discussed above have provided valuable mechanistic and phenotypic insights while providing a robust platform for testing gene therapy strategies. However, the ability to differentiate human adult stem cells *in vitro* in order to generate “retina-in-a-dish” and “retinal disease-in-a-dish” models has created several exciting new opportunities. First, these models provide a viable alternative to animal models for addressing basic mechanistic questions regarding ocular morphogenesis, for example by modulating gene expression in optic vesicles from patient-derived induced pluripotent stem cells (iPSCs) (Capowski et al., 2016). Second, assays to measure transgene expression and biological activity can be developed using knockout iPSC-derived retinas (Quinn et al., in press). Third, these models can be used both to test gene-editing strategies and for high-throughput drug screening. Finally, these models can serve as a source of transplantable material for cell therapy strategies. In all of these applications, the material used will be based on human cells and is disease-specific. Many studies using rodent and/or primate models have shown that photoreceptor cell transplantation is a feasible strategy for improving retinal function (Lamba et al., 2009; Pearson et al., 2012; Gonzalez-Cordero et al., 2013; Jayaram et al., 2014; Shirai et al., 2015). Recently, donor-host cytoplasmic exchange was highlighted as a major pathway used by transplanted photoreceptors alongside the classically depicted processes of migration and integration. Because this transfer of cytoplasmic material between donor and host photoreceptors is not due to classic cell fusion or facilitated uptake from the extracellular matrix, it may represent a new therapeutic strategy for use in retinal disease (Pearson et al., 2016; Santos-Ferreira et al., 2016; Singh et al., 2016).

Of course, despite their advantages these *in vitro* models have several possible shortcomings. For example, the *in vitro* retina-in-a-dish model lacks the full macroscopic environment of the entire organism. In addition, these techniques are time-consuming and costly, including the need to generate knockout and/or patient iPSCs which then need to differentiate and mature to form functional retina-like or diseased retina-like structures. Generating retina-like organoids from human embryonic stem cells and iPSCs is relatively autonomous, although neural induction requires the addition of extrinsic factors such as B-27 and N-2 supplements. However, providing additional factors such as retinoic acid and Notch inhibitors can accelerate neuronal development and maturation (Wiley et al., 2016). The use of *in vitro* disease models using human iPSCs has begun to overtake the use of human embryonic stem cells, due in large part to ethical concerns and technical issues (Zacharias et al., 2011). It is also interesting to note that the *in vitro* model mimics well the *in vivo* development. Mouse optic vesicles develop a fully layered neural retina in just a few weeks; in contrast, human optic vesicles take at least 180 days to develop a neural retina with yet immature photoreceptor segments (Zhong et al., 2014). Therefore, mouse iPSC-derived retinas may be applicable for more basic, high-throughput initial testing, although differences in retinal photoreceptor composition between species should be considered. A more recent method developed for differentiating cells is the self-formed ectodermal autonomous multi-zone. This method mimics the development of the entire eye by differentiating cells into four principal zones to recreate the retinal pigment epithelium, retina, lens, and ocular surface ectoderm (Hayashi et al., 2016). This method may be more suitable for cell-based correction and transplantation, as well as for use in patients with a disease that involves multiple ocular tissues.

## Personalized medicine: still not yet the ideal situation

The development of a proof-of-concept therapy for a gene linked to a retinal disease will likely be driven by technological advances that lead to a more streamlined approach in order to realize “personalized medicine.” The recent advent of gene-editing and gene-replacement strategies, improved cell targeting, the ability to package genes into delivery vectors, and *in vitro* models has certainly helped reduce the time needed to obtain the first proof-of-concept results for other gene-linked retinal diseases. Over the past several years, the development of “retinal disease-in-a-dish” modeling approaches has led to a highly robust and widely used treatment development pipeline that spans from patient identification to therapy. Several groups are now focusing their efforts on improving this pipeline further in order to streamline the *in vitro* process, providing several important advantages. First, new, less invasive sources of human iPSCs become available, providing more efficient generation of these iPSCs. In practical terms, this means that iPSCs can be obtained from blood, urine, and dermal pulp samples, as an alternative to skin biopsies; this is particularly beneficial in children (Loh et al., 2010; Beltrão-Braga et al., 2011; Valamehr et al., 2012; Zhou et al., 2012). Second, patient phenotyping can be improved through the use of disease models and transcriptomics, providing greater insight into the underlying pathway dynamics. Third, optimal human retinal-disease-in-a-dish procedures allow improved treatment paradigms for the patient (Kaewkhaw et al., 2016; Völkner et al., 2016). Lastly, this approach allows researchers to develop strategies designed to correct point mutations and exon insertions in both dividing and non-dividing neurons using CRISPR/Cas9-based editing (Bassuk et al., 2016; Suzuki et al., 2016).

**Figure 6:**
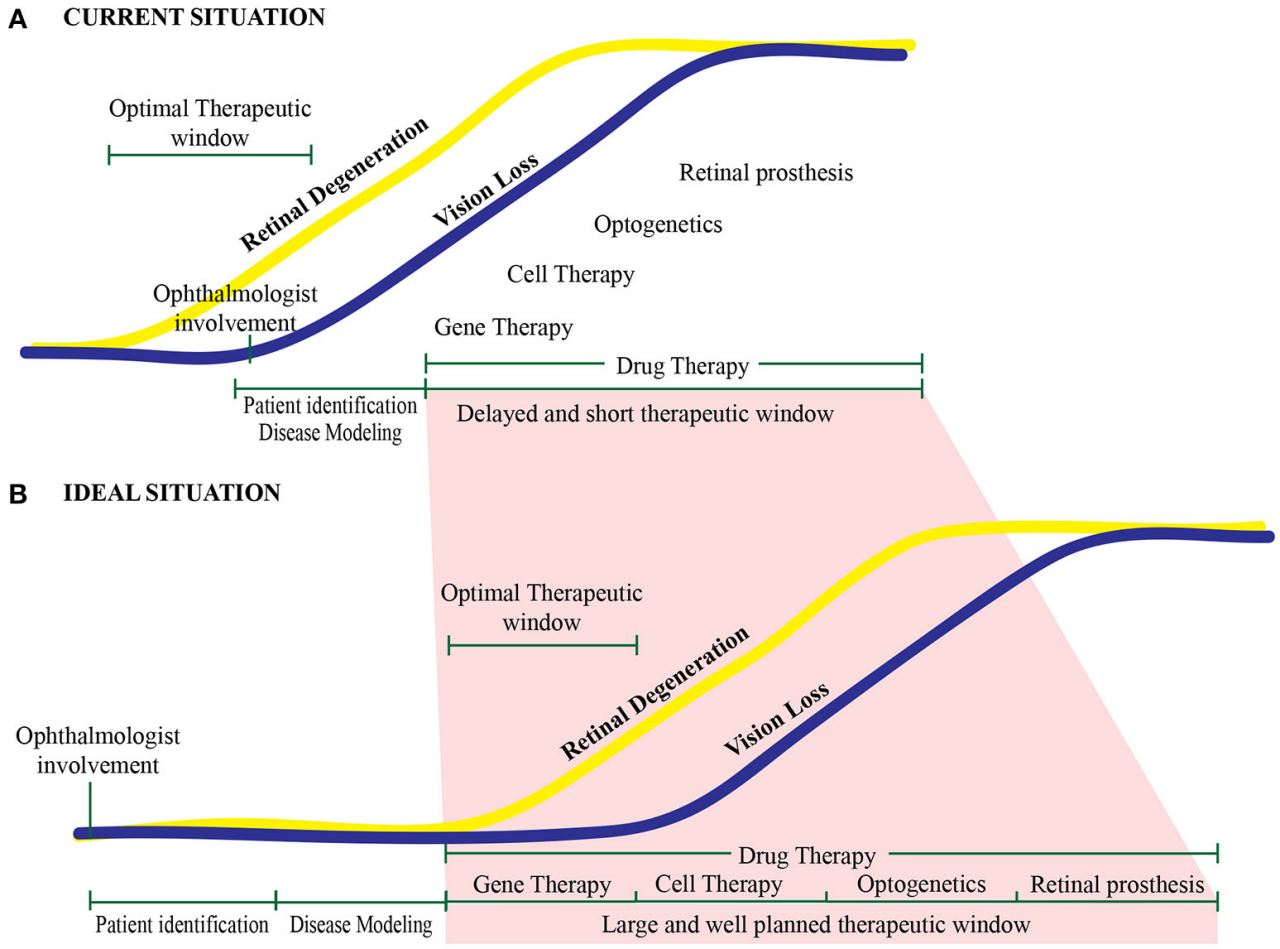
**Proposed therapeutic timeline for treating retinal diseases. (A)** With current approaches, the optimal therapeutic window is missed in most patients. Typically, an ophthalmologist becomes involved—and treatment paradigms are initiated—only after the onset of retinal degeneration and vision loss. **(B)** Under ideal conditions, a patient at risk for developing an inherited retinal degeneration will be identified well before disease onset and the start of vision loss. This will enable the clinician to intervene within the therapeutic window, providing a well-planned, personalized intervention.

In a typical clinical situation, patients are identified, screened, and given a diagnosis only after retinal degeneration has already begun. Thus, the optimal therapeutic window may have already closed by this time (Figure 6A). Delaying diagnosis can affect the therapeutic window, reducing the efficacy of potential gene therapies, ultimately reducing patient outcome. In this respect, other therapeutic strategies such as cell transplantation, optogenetics, and the use of a retinal prosthesis might be more applicable. In the future, this will hopefully become less of an issue as we understand better the pathophysiology of retinal diseases and as treatment platforms become routine practice. In the ideal scenario, a patient with a putative hereditary retinal disease will seek out an ophthalmologist in order to obtain a diagnosis and genetic screening before the onset of vision loss. In addition, the use of *in vitro* “retinal disease-in-a-dish” approaches—in which the cultured retina is physiologically stressed—will likely lead to earlier identification of the retinal phenotype in prospective patients, ultimately providing a more structured approach to developing and implementing gene therapies (Figure 6B). After clinical studies using degenerated retinas demonstrate therapeutic efficacy, this early-stage planning may also increase the rate of success by providing treatment at the optimal time during disease progression.

## Retinal gene therapy and CRISPR/Cas9

In recent years, gene therapy has been used successfully to demonstrate the viability of providing therapeutic—albeit transient—benefits. Current clinical trials for the *RPE65, REP1*, and *CNGA3* genes have revealed both the effectiveness and limitations associated with retinal gene therapy, including the timing, injection method, and transduction coverage (Bainbridge et al., 2015; Edwards et al., 2016; Fischer et al., 2016; https://clinicaltrials.gov; Maguire et al., 2008). However, these limitations do not necessarily suggest that gene therapy will not be able to halt the degenerative process, except perhaps at a much later stage in the disease (Cepko and Vandenberghe, 2013; Cideciyan et al., 2013; Koch et al., 2015; Hurley and Chao, 2016). These technical limitations will likely require a more technological advance than simply reinventing the wheel. While gene-augmentation therapies are currently the most used and most validated strategy, gene editing—in which the faulty gene is replaced with a healthy copy—is potentially more appropriate, as it corrects the specific genetic defect within the endogenous gene. In recent years, the CRISPR/Cas9 approach has largely replaced previous gene-editing methods, including transcription activator-like effector nucleases and zinc finger nucleases, and several research groups are currently competing to establish proof-of-concept in the retina.

CRISPR/Cas9 is a bacterial defense system in which Clustered Regularly Interspaced Short Palindromic Repeats (CRISPR) allow the identification of previously invaded viruses. Upon binding with a Cas (CRISPR-associated) protein, the resulting complex then drives the cleavage of DNA in the invading virus. Artificially synthesized guide RNA can be used together with a Cas protein to induce double-strand breaks in the target gene. Despite its growing popularity, however, the CRISPR/Cas9 system is not perfect, as the guide RNA can bind to similar sites outside of the targeted gene, potentially leading to unspecified and unintended mutations, thus limiting both its research value and clinical potential (Fu et al., 2013). Nevertheless, CRISPR/Cas9 has been used to correct defects in several genes, including genes linked to Duchenne muscular dystrophy, metabolic liver disease, and hemophilia B (Guan et al., 2016; Long et al., 2016; Maggio et al., 2016; Nelson et al., 2016; Tabebordbar et al., 2016). Correcting a point mutation requires that the Cas9 protein, guide RNA, and donor template for recombination are introduced together into the same cells. This strategy has been used successfully in patient-specific iPSCs to repair a point mutation in the *RPGR* gene associated with X-linked retinitis pigmentosa (Bassuk et al., 2016). However, to apply this strategy *in vivo* currently requires a double-AAV delivery system, with one AAV containing Cas9 and the other AAV containing the guide RNA and donor template; thus, packaging everything into a single delivery vector is the next challenge (Yang et al., 2016). Another major—albeit recently solved—drawback associated with this method is that it must be used in dividing cells. Of course, early treatment of the diseased retina would be ideal, but ethically this will likely not become possible until safety and regulatory hurdles are overcome. In this respect, obtaining proof-of-concept in both *in utero*-treated mouse models and *in vitro* iPS-derived human disease models may help facilitate this process. Proof-of-concept has already been demonstrated for genomic editing in non-dividing photoreceptors using *in vivo* CRISPR/Cas9-mediated homology-independent targeted integration. Using the Royal College of Surgeons (RCS) rat model of retinitis pigmentosa, the authors showed both an improved morphological outcome and an improved electroretinography response (Suzuki et al., 2016). With respect to developing a cell therapeutic approach for use in later stages of degeneration, CRISPR/Cas is a potentially viable method, particularly with the off-target effects being minimized using more specific guide RNAs and an array of other, recently discovered endonucleases such as Cpf1 (Fu et al., 2014; Zetsche et al., 2015, 2017). In summary, at least for the foreseeable future, complete gene replacement using gene-augmentation strategies appears to be the most viable and validated therapeutic strategy for inherited retinal degenerations.

## Is targeting *CRB* a feasible gene therapy approach?

**Figure 7:**
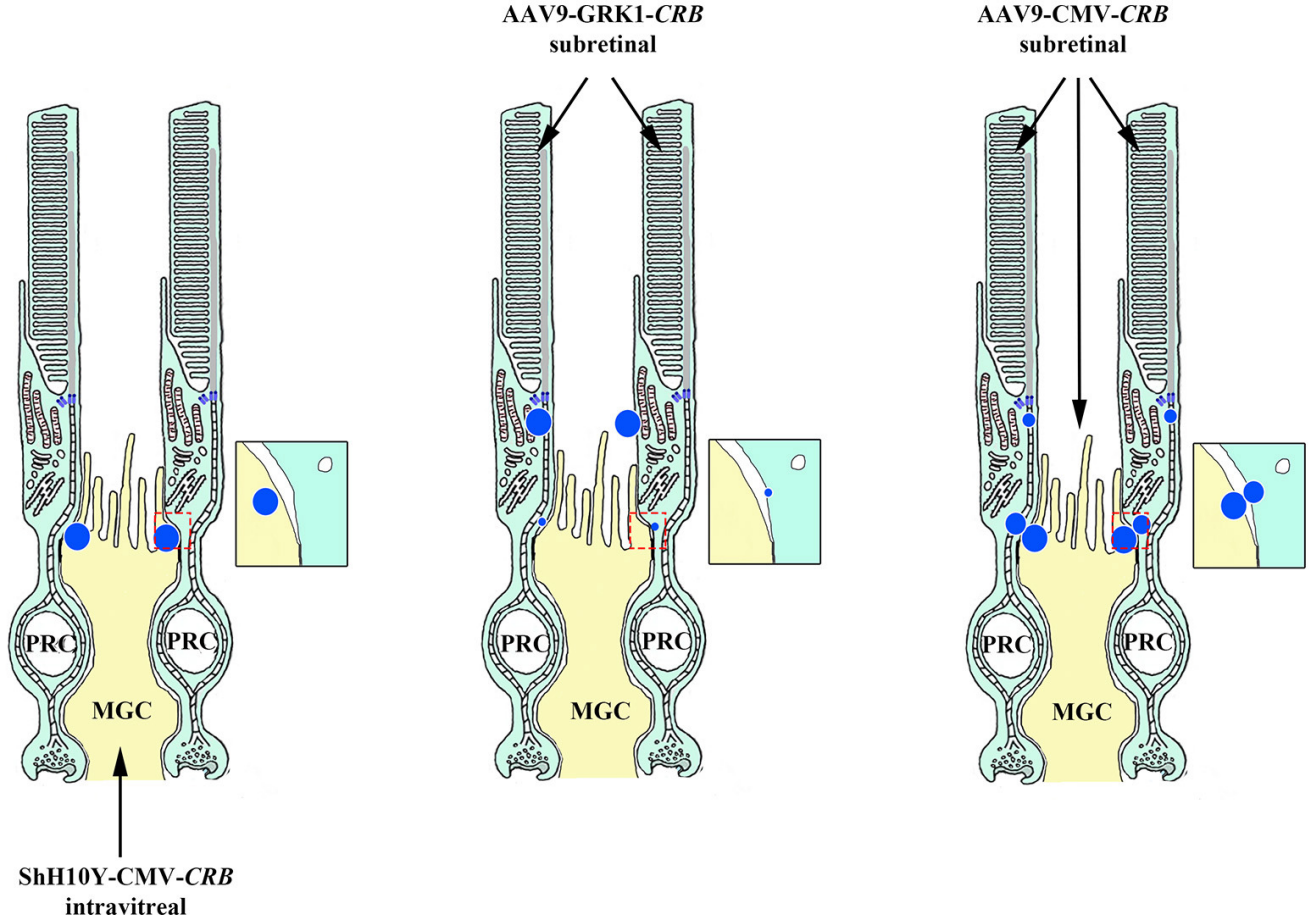
**Schematic depiction of ***CRB***-mediated gene therapy strategies**. Targeted delivery of CRB exclusively to either the Müller glial cells (MGC) or photoreceptors (PRC) provides no therapeutic benefit (left and middle panels, respectively); in contrast, delivering CRB to both MGCs and PRCs (right panel) elicits a response. Intravitreal applied ShH10Y-CMV-*CRB* drives CRB expression in the subapical region (SAR) of MGCs, whereas subretinal applied AAV9-GRK1-*CRB* drives expression at the SAR of photoreceptors. In contrast, subretinal applied AAV9-CMV-*CRB* drives expression at the SAR of both cell types.

The feasibility of using a *CRB*-based gene therapy approach seems to depend upon the ability to restore pre-disease levels of CRB expression in order to sufficiently stop the degeneration process. However, unlike other therapies, this approach may not be as simple as replacing one gene for a similar gene, nor as simple as targeting the gene replacement to a single cell type. Although the *CRB1* gene was first linked to retinal disease back in in 1999, it took 16 years to obtain the first *in vivo* proof-of-concept for *CRB1*-based gene therapy. This long interval was due in part to several factors, including: (i) the sheer size of the *CRB* gene sequences, which limited their ability to be packaged in AAV vectors, (ii) the need to engineer vectors with codon optimization, and (iii) the need to develop minimal promoters in order to express CRB proteins in Müller glial cells and photoreceptors (Pellissier et al., 2014a). Expressing the human *CRB1* gene in mutant *Crb1* mouse models—but not in wild-type mice—led to an adverse immune response (Pellissier et al., 2015). It is possible that some *CRB1* mutations lead to nonsense-mediated mRNA decay, leaving these patients immunologically susceptible to the expression of recombinant human CRB1 protein. In these patients, T cells primed against the human wild-type CRB1 protein would be activated by the new CRB1 epitopes on the surface of antigen-presenting cells, inducing an immunogenic response. To circumvent this problem, the most structurally similar CRB member—CRB2—was expressed at near physiological levels. Expressing human CRB2 in the retina of mice expressing normal levels of the mouse homologs had no discernible detrimental effects. Importantly, overexpressing human CRB2 in photoreceptors and Müller glial cells with reduced levels of endogenous Crb2 and Crb1 expression improved both cell morphology and retinal activity, and the human CRB2 protein was expressed at the appropriate subapical regions; interestingly, expressing human CRB2 in only one cell type had no effect. This supports our finding that adequate levels of CRB protein in only a single cell type is insufficient for maintaining retinal integrity (Figure 7; Pellissier et al., 2015). It is also important to ensure that the CRB2 protein is localized correctly at the subapical region when expressed in both photoreceptors and Müller glial cells. When expressed only in photoreceptors, CRB2 localized at the tip of the inner segments at higher levels than in the subapical region (Pellissier et al., 2015). This highlights the need for CRB to be expressed in both Müller glial cells and photoreceptors and to localize correctly to the subapical region, thereby promoting the maintenance of adherens junctions via the cadherin-catenin complex. In addition, this underscores our current lack of knowledge regarding the physiological relevance of CRB homomeric and perhaps heteromeric interactions via their extracellular domains. Although these CRB-mediated cell-cell interactions are poorly understood in mammals, homomeric interactions between Crb2 extracellular domains in zebrafish photoreceptors have been suggested to promote cell-cell adhesion (Zou et al., 2012). In summary, although Müller glial cell-Müller glial cell interactions and photoreceptor-photoreceptor interactions alone are likely not sufficient for maintaining retinal structure and function in patients with *CRB1*-linked mutations, Müller glial cell-photoreceptor interactions may be sufficient.

This brings us to the clinically relevant question. Given that the human retina contains significant levels of CRB2 in Müller glial cells, would *CRB2*-mediated gene therapy specifically targeted at photoreceptors be sufficient to rescue function in patients, or will the levels of CRB2 in Müller glial cells also need to be increased? As discussed above, the levels of functional CRB1 protein are reduced in Müller glial cells and photoreceptors in patients with mutations in the *CRB1* gene. The question remains, will increasing CRB2 expression in photoreceptors be sufficient to restore the properties of CRB-CRB-mediated Müller glial-photoreceptor interactions as in healthy persons, and will this mimic the CRB2-CRB2-mediated Müller glial cell-photoreceptor interactions observed in retinal CRB1-deficient mice and zebrafish (which develop late-onset retinal degeneration and no retinal degeneration, respectively). This train of thought gives rise to reservations regarding moving forward with human *CRB1*-directed therapy targeted to both cell types (although this strategy might be a viable option for a specific subset of patients who lack T cells directed against CRB1). Given the high levels of both structural and functional overlap between CRB1 and CRB2, as well as the apparent need to express CRB proteins in both photoreceptors and Müller glial cells in order to maintain a functional retina, we believe that human *CRB2*-mediated gene therapy may represent a safe and viable treatment for fighting blindness due to mutations in *CRB1*.

## Future developments

Thanks to the array of mouse models currently available for addressing questions regarding CRB function and protein interactions, together with the proof-of-concept showing the feasibility of gene therapy, we now have a number of tools at our disposal to help launch *CRB*-mediated therapy into preclinical trials, ideally in the near future. Moreover, several cutting-edge methods and techniques are now available, including: (i) CRISPR/Cas9, to correct specific point mutations in patients; and (ii) the ability to differentiate human iPSCs in order to generate humanized retinal models for investigating the pathways that underlie retinal disease, to test vector-mediated gene therapies using potency assays, and to serve as a viable source of transplant tissue. Together, these powerful new technologies will accelerate the field toward developing treatment options and addressing fundamental questions.

## Author contributions

All authors have made substantial, direct, intellectual contributions to the work, and all authors approve the publication of this manuscript.

### Conflict of interest statement

The LUMC is the holder of patent number PCT/NL2014/050549, which describes the potential clinical use of CRB2; JW and LP are listed as inventors on this patent, and JW is an employee of the LUMC. The authors declare that the research was conducted without any commercial or financial relationships that could be construed as a potential conflict of interest.
